# Using deep DenseNet with cyclical learning rate to classify leukocytes for leukemia identification

**DOI:** 10.3389/fonc.2023.1230434

**Published:** 2023-09-12

**Authors:** Essam H. Houssein, Osama Mohamed, Nagwan Abdel Samee, Noha F. Mahmoud, Rawan Talaat, Aymen M. Al-Hejri, Riyadh M. Al-Tam

**Affiliations:** ^1^ Faculty of Computers and Information, Minia University, Minia, Egypt; ^2^ Faculty of Computers and Artificial Intelligence, Beni-Suef University, Beni-Suef, Egypt; ^3^ Department of Information Technology, College of Computer and Information Sciences, Princess Nourah bint Abdulrahman University, Riyadh, Saudi Arabia; ^4^ Rehabilitation Sciences Department, Health and Rehabilitation Sciences College, Princess Nourah bint Abdulrahman University, Riyadh, Saudi Arabia; ^5^ Biotechnology & Genetics Department, Agriculture Engineering, Ain Shams University, Cairo, Egypt; ^6^ School of Computational Sciences, Swami Ramanand Teerth Marathwada University, Nanded, Ma-harashtra, India

**Keywords:** leukemia, leukocytes, DenseNet, transfer learning, cyclical learning rate

## Abstract

**Background:**

The examination, counting, and classification of white blood cells (WBCs), also known as leukocytes, are essential processes in the diagnosis of many disorders, including leukemia, a kind of blood cancer characterized by the uncontrolled proliferation of carcinogenic leukocytes in the marrow of the bone. Blood smears can be chemically or microscopically studied to better understand hematological diseases and blood disorders. Detecting, identifying, and categorizing the many blood cell types are essential for disease diagnosis and therapy planning. A theoretical and practical issue. However, methods based on deep learning (DL) have greatly helped blood cell classification.

**Materials and Methods:**

Images of blood cells in a microscopic smear were collected from GitHub, a public source that uses the MIT license. An end-to-end computer-aided diagnosis (CAD) system for leukocytes has been created and implemented as part of this study. The introduced system comprises image preprocessing and enhancement, image segmentation, feature extraction and selection, and WBC classification. By combining the DenseNet-161 and the cyclical learning rate (CLR), we contribute an approach that speeds up hyperparameter optimization. We also offer the one-cycle technique to rapidly optimize all hyperparameters of DL models to boost training performance.

**Results:**

The dataset has been split into two sets: approximately 80% of the data (9,966 images) for the training set and 20% (2,487 images) for the validation set. The validation set has 623, 620, 620, and 624 eosinophil, lymphocyte, monocyte, and neutrophil images, whereas the training set has 2,497, 2,483, 2,487, and 2,499, respectively. The suggested method has 100% accuracy on the training set of images and 99.8% accuracy on the testing set.

**Conclusion:**

Using a combination of the recently developed pretrained convolutional neural network (CNN), DenseNet, and the one fit cycle policy, this study describes a technique of training for the classification of WBCs for leukemia detection. The proposed method is more accurate compared to the state of the art.

## 1 Introduction

Medical images are a massive data source for the healthcare sector. With developments in imaging technology and processing capabilities, the demand for increasingly complex tools to interpret images has developed. More accurate image analysis will save healthcare costs and improve the quality of diagnosis, ultimately leading to better patient outcomes. Anemia, leukemia, and malaria are just a few of the blood disorders that can be detected with improved pathologists’ ability to recognize, count, and classify blood cells (1–3). Improved understanding will facilitate treatment, reduce potentially dangerous drug interactions, and facilitate health monitoring. The three types of cells that make up human blood are the erythrocytes (red blood cells), leukocytes (white blood cells (WBCs)), and thrombocytes (platelets). All three are derived from lymphoid and bone marrow stem cells. Erythrocytes, which are non-nucleated biconcave diskettes, transport both carbon dioxide (CO_2_) and oxygen (O_2_) around the body. Blood is composed of roughly 40%–45% red blood cells and 1% WBCs (4–6). Organs in the body rely on each of the three types of blood cells for specific tasks. Nevertheless, WBCs are made in the bone marrow and are a crucial part of the blood’s immune system. The immune system is the body’s primary line of defense against invaders, most notably pathogens, and is mostly the work of WBCs (7).

Thrombocytes, often known as platelets, are smaller than erythrocytes and lack a nucleus. Giemsa staining produces a vivid purple tint in platelets (8). Platelets are crucial to the body’s clotting process, which guards against bacterial invasion and keeps the body from bleeding out continuously following injuries (9). Leukocytes may be divided into five major types based on a variety of characteristics, including cell size, nucleus shape, type of nucleus lobes, granule cytoplasm-to-nucleus ratio (CNR) staining qualities, and function.

**Figure 1:**
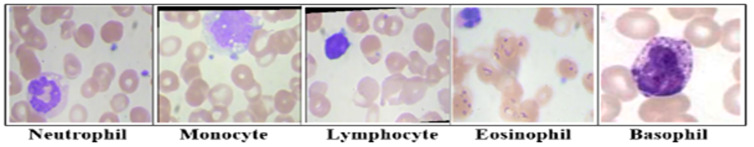
The main types of leukocytes cell images. Lymphocytes, Monocytes, Neutrophils, Eosinophils, and basophils are the five most common types of white blood cells. Each type has a certain nucleus shape.

Lymphocytes, monocytes, neutrophils, eosinophils, and basophils are the five most common types of WBCs. Another thing is the band identification for a certain nucleus shape. **Figure 1** illustrates several common types of leukocytes. A decrease in leukocytes below the threshold is medically referred to as leukopenia. It is evidence of the frailty of the immune system and a potential reason for disease.

Leukocyte counts can be low for one of two major reasons: either the bone marrow has ceased producing leukocytes or an infection is present and causing cells to be destroyed more quickly than they can be replaced. Leukocytosis, a proliferative condition, is characterized by a rise of leukocytes over the upper limit, which is typically an indication of an inflammatory reaction. It occasionally takes place because of normal immunological responses. Nonetheless, if the neoplasm has an abnormally high or low cell count, or if autoimmunity causes immunological reactions, it will be classified as abnormal. Leukocyte disorders can also be classified in this fashion (10) based on the nature and function of affected cells. Hematologists can discover a great deal about blood diseases such as anemia, bleeding disorders, leukemia, and HIV positivity from a complete blood count (CBC) and differential blood count (DBC). The CBC can be performed automatically by a cytometer as blood flows past the detector, with parameters including hematocrit and hemoglobin measured (11). DBC, which may count the different types of leukocytes in peripheral blood, was previously performed by a blood pathologist physically inspecting blood smears under a light microscope. Nonetheless, this process is sensitive, and it is essential that there be no (or just very few) inspection errors made by the human professional. However, after several hours of examination, specialists might often feel exhausted and make false identifications of the various WBCs. This can happen rather frequently (3, 12). As a result of the development of both theoretical and practical applications for the technology that is available today, several different methods of blood analysis that are either fully or partially automated and are based on the image analysis of blood smears or the principles of flow cytochemistry have been developed. Image processing and artificial intelligence (AI) (13) have lately been used to develop several new methods that researchers have designed to automate the leukocyte classification process. Within the scope of this investigation, a fully automated computer-aided diagnosis (CAD) system of leukocytes has been developed and implemented. The proposed CAD system includes four primary stages, which are the image preprocessing and enhancement stage, the image segmentation stage, the feature extraction and selection stage, and the WBC classification stage.

**Figure 2:**
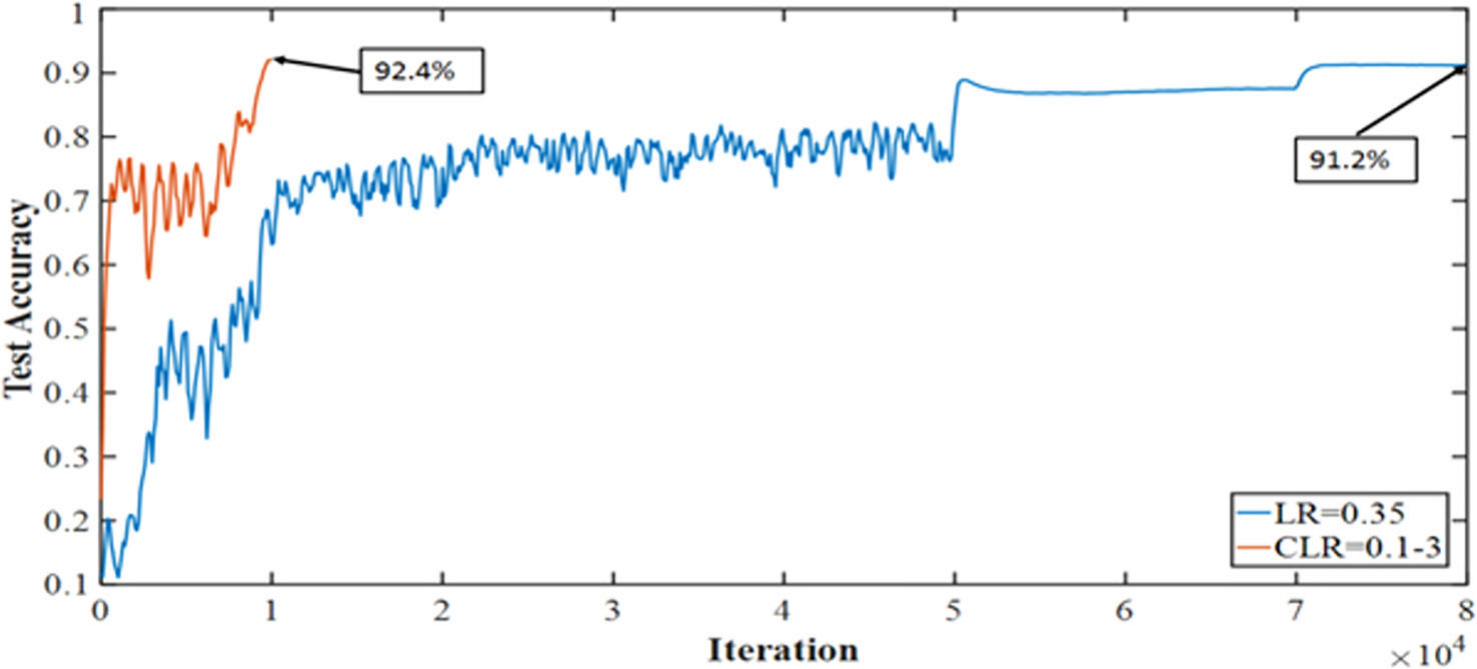
Accuracy achieved with one cycle of training against the conventional method of training Convolutional Neural Networks.

The medical imaging industry makes extensive use of the recently developed and powerful pretrained convolutional neural network (CNN) DenseNet-161. However, compared to other pretrained CNNs, it has a high processing time and cannot generalize. Thus, we are exploring the one cycle policy (14, 15), a technique used to shorten training time while simultaneously enhancing performance and tuning all hyperparameters of deep learning (DL) models (15, 16). As can be shown in **Figure 2**, a cyclical learning rate (CLR) can produce better training results than the default learning rate (LR).

In contrast to blood cell segmentation algorithms that rely on watershed segmentation, this article presents a segmentation algorithm that uses Bounded Opening followed by Fast Radial Symmetry (BO-FRS)-based seed-point detection and hybrid Ellipse Fitting (EF)-based contour estimation. These methods accurately extract seed points and precisely segment overlapping cells, even from low-contrast inhomogeneous visual features. This makes the method suitable for complex blood cell segmentation problems. The proposed Least Squares (LS)-based geometric ellipse fitting approach leads to better accuracy (ACC) and more localization compared to algebraic Ellipse Fitting Methods (EFMs), which are prone to biased fitting parameters and inaccurate boundaries. The proposed method combines the benefits of geometric and algebraic EFMs and is computationally efficient. It also solves the noise problem with an Laplacian of Gaussian (LoG)-based modified high-boosting operation and avoids oversegmentation. This approach can also be applied to other medical applications such as MRI, CT, ultrasound, and X-ray images, as well as cybernetic applications and the segmentation of overlapping objects. Notably, the proposed algorithm does not require training data, making it more suitable than DL-based techniques when little or no data are available for training (17).

The following is a list of the contributions that were made to bring attention to the significance of the work that we will be presenting:

- We present an improved, lightweight, and effective CAD system that can automatically classify four types of leukocytes (neutrophils, eosinophils, lymphocytes, and monocytes), which is a significant contribution to the field of medical image analysis.
- We investigate the potential of DenseNet-161 pretrained CNN for the suggested CAD system, which is a modern approach to developing the system.
- The authors train the DenseNet efficiently with a single cycle policy, cutting down on epochs and iterations, and thereby making use of big datasets. This is a significant contribution to the field of DL, as it demonstrates a more efficient approach to training CNNs.
- The proposed model is tested experimentally on a variety of real-world datasets, which is a significant contribution to the field of medical image analysis, as it demonstrates the effectiveness of the model on a range of different datasets.
- The results of the study show that the proposed model outperforms the gold standard classification model, which is a significant contribution to the field of medical image analysis.
- The achieved ACC in categorization is approximately 99.8%, which is a significant contribution to the field of medical image analysis, as it demonstrates the high ACC of the proposed model.

The sections of this paper are as follows: Section 2 (Literature Review) details the related work. In Section 3 (Materials and Methods), we provide some the datasets and methods utilized for the proposed model. The analysis and results of the experiments are presented in Section 5 (Results and Discussion). In the end, the paper was concluded in Section 6 (Conclusion).

## 2 Literature review

Many attempts at automatically segmenting, categorizing, and analyzing leukocytes have been published. The automatic analysis of medical images such as microscopic blood smears has attracted the attention of many researchers. Numerous scientists have argued for employing machine learning (ML) and AI to automatically detect and diagnose abnormalities in microscopic images of leukocytes. CAD of leukocytes can be broken down into two categories: those that use ML (18) and those that use DL (19). Both ML and DL are described and summarized here. **Table 1** provides a summary of DL-based methods and serves to contrast our proposed work with the state-of-the-art DL-based methods. **Table 1** summarizes the current state of the field and the limitations of each technique based on recent studies that employed DL algorithms to identify abnormalities in leukocytes. The most noteworthy aspects of the new system are highlighted in the table together with the results of the performance evaluation in terms of ACC.

**Table 1:** Overview of research using DL techniques for leukocyte classification or segmentation.

Author	Method	Accuracy	Dataset	Data volume
Maryam et al. (20)	Optimized CNN	99%	BCCD	12,444
Bani–Hani et al. (21)	GA-optimized CNN	91%	BCCD	12,435
Liang et al. (22)	Hybrid CNN-RNN	90.80%	BCCD	12,444
Rao (23)	CNN and ResNeXt	99.24%	BCCD	12,444
Rao (24)	ANN and CNN	97.70%	BCCD	1,600
Baydilli, and Atila (25)	Capsul Networks	96.90%	LISC	263
Ghosh and Bhattacharya (26)	CNN and FCN on noise-free cell images	98.40%	BCCD	12,500
Wang et al. (27)	Single Shot Multibox Detector and YOLO	90.09%	Private database	11,600
Ma et al. (28)	DC-GAN, and ResNet	91.70%	BCCD	12,447
banik et al. (29)	CNN	96%	BCCD	12,811
Sahlool et al. (30)	VGGNet, CNN, and SESSA	83.20%	C-NMC	10,661
Proposed model	DenseNet with Cyclical Learning Rate	99.80%	BCCD	12,447

CNN, Convolutional Neural Network; GA, Genetic Algorithms; ANN, Artificial Neural Network; FCN, Fully Connected Network.

The following studies represent leukocyte diagnosis research that has been conducted using classical ML. Sanei et al. (14) have utilized the Bayesian classifier for the classification of leukocytes. They have split the blood microscopic image into three sections. Instead of relying on the image’s geometric or physical properties, they used a Bayesian classifier to isolate the Eigen cells. Decisions were based on the relative density of various colors. First, the input photographs were rescanned, segmented, and rotated, and the three vectors representing intensity and color were identified. Leukocyte images from 10 patients were employed by Sarrafzadeh et al. (31), who trained a support vector machine (SVM) using a set of parameters that includes six geometrical qualities, six color attributes, six statistical features, and seven-moment invariances (invariants). The classifier reported an ACC rate of over 93%. Leukocyte borders in images are defined manually to reduce the impact of segmentation errors. The cytoplasm and nucleus of leukocytes were separately identified by the Fuzzy C-means clustering method. Thereafter, the cytoplasm, nucleus, and other components of the cell that are of interest are removed Ko et al. (32) used SVMs to classify the 480 blood smear images into training and testing sets. They claimed that random forest performed better than multilayer SVM when it came to classification. In a previous work, the snake algorithm has been utilized to divide leukocytes. They used the shape, color, and texture of the image as criteria for classification. Gaussian normalization was then utilized to transform the feature vectors from 0 to 1 after feature extraction (32). Ramoser et al. employed SVM to automatically grade leukocytes. The study of 1,166 images split into 13 categories found that segmentation was performed with 95% ACC (94/100) and classification was performed with 75%–90% ACC. In their study, Theera-Umpon and Dhompongsa (33) analyzed if it was possible to classify leukocytes using only data from their nuclei. To prevent segmentation errors from affecting the results of the investigations, the cell nuclei were removed manually. Bayes classifiers and CNNs were used for classification. They determined that the information obtained from cell nucleus 100 was adequate because their classification was correct 77% of the time. WBC subtype detection by flow cytometry was proposed by Adjouadi et al. Parametric datasets were analyzed in a multidimensional space using SVMs (34). To classify WBCs, Rodrigues et al. created a two-stage artificial neural network. To reduce the 106 problems, they first employed the Back Propagation Neural Network (BPNN) for preclassification and then presented a hybrid model based on the SVM and the pulse-coupled neural network (PCNN). As a result, they looked for ways to lessen the negative effects (35).

Both Otsu’s automated thresholding methodology and the image enhancement and arithmetic strategy were proposed by Joshi et al. for separating leukocytes from red blood cells. The K Nearest Neighbor (K-NN) classifier was used to separate blast cells from typical lymphocyte cells. Their ACC was determined to be 93% based on the results of the tests (36). Image processing methods were used by Tantikitti et al. (37) for classifying WBCs, extracting features from edges, changing colors, and fragmenting images. Patients with dengue virus infections were sorted using a decision tree analysis. The results showed that a total of 167 cell shots were able to accurately classify leukocytes (92.2% ACC) and that 264 blood cell photos correctly classified dengue (72.3%). One hundred fifteen images were used by Hiremath et al. (38) as input parameters for AI-based algorithms that categorized WBCs based on their color, texture, and geometric properties. Histogram equalization, edge extraction, and threshold-based automatic segmentation for lymphocytes, monocytes, and neutrophils are the focus of that study. Several images of blood smears were used in the trials, with geometric features of the images being utilized in the classification process. Habibzadeh et al. (39) employed the shape, density, and texture of microscopic images of blood to classify and count leukocytes. The parameters of the SVM classifier were the wavelet characteristics that were generated for the classification process using the dual-tree complex wavelet transform (DT-CWT) approach.

Ramesh et al. (40) proposed a simple classification method that incorporates morphological characteristics and color data. As the first step in a two-stage classification process, leukocyte cell nuclei and leukocyte boundaries have been meticulously established. The second stage involved applying the linear discriminant analysis method to implement the features found in the cytoplasm and nucleus of leukocytes. In another study, Su et al. (41) classified leukocytes into five distinct groups, each with its own set of distinguishing features. In this location, they aimed to use morphological mechanisms to segment the elliptical nuclei and cytoplasm of leukocytes. These photo chunks were mined for geometric elements, color characteristics, and texture qualities based on LDP (local directional pattern) and then used to train three distinct neural networks. For the testing, they used 450 images of leukocytes, and the highest identification ACC was 99.11%.

The microscopic analysis of blood cells is crucial for the early diagnosis of life-threatening hematological disorders such as leukemia. This paper presents an effective and computationally efficient approach for automatically detecting and classifying acute lymphoblastic leukemia (ALL) and acute myeloid leukemia (AML). Das et al. (42) proposed an approach that uses transfer learning, which has been successful in medical image analysis due to its excellent performance in small databases. The proposed system employs a lightweight transfer learning-based feature extraction followed by SVM-based classification technique for efficient ALL and AML detection. The system is faster and more efficient due to the depthwise separable convolution, tunable multiplier, and inverted residual bottleneck structure. Moreover, the SVM-based classification technique improves the overall performance by optimizing the hyperplane location. The experimental results demonstrate that the proposed system outperforms others in all three publicly available standard databases, including ALLIDB1, ALLIDB2, and ASH.

Breast cancer is a leading cause of cancer-related deaths among women worldwide, and early detection is crucial for successful treatment. In this work, the authors have developed five new deep hybrid CNN-based frameworks for breast cancer detection. Sahu et al. (43) proposed that hybrid schemes exhibit better performance than the respective base classifiers by combining the benefits of both networks. A probability-based weight factor and threshold value are essential for efficient hybridization. An experimentally selected optimum threshold value makes the system faster and more accurate. Notably, unlike traditional DL methods, the proposed framework yields excellent performance even with small datasets. The proposed scheme is validated with datasets of breast cancer: mini-DDSM (mammogram), BUSI, and BUS2 (ultrasound). The experimental results demonstrate the superiority of the proposed ShuffleNet-ResNet scheme over the current state-of-the-art methods in all of the mentioned datasets. Moreover, the proposed scheme achieves high ACC rates of 99.17% and 98.00% for abnormality and malignancy detection in mini-DDSM, respectively, and 96.52% and 93.18% for abnormality and malignancy detection in BUSI, respectively. In BUS2, the proposed scheme delivers 98.13% ACC for malignancy detection.

Sahu et al. (44) introduce a breast cancer detection framework based on DL that utilizes EfficientNet to achieve high performance even in cases of small databases. The framework incorporates uniform and adaptive scaling of depth, width, and resolution to ensure an optimal balance between classification performance and computational cost. Furthermore, a Laplacian of Gaussian-based modified high boosting (LoGMHB) is employed as a preprocessing step, along with data augmentation, to enhance the system’s performance. The study evaluated the proposed method on mammogram and ultrasound modalities and demonstrated its superiority over other methods in all performance measures. The experimental results were obtained using 5-fold cross-validation and showed promising results for automatic and accurate detection of breast cancer at an early stage, which could lead to proper treatment and greatly reduce mortality rates.

The early detection of leukemia is crucial for proper treatment planning and improving patient outcomes. Microscopic analysis of WBCs is a cost-effective and less painful approach for detecting leukemia. However, automatic detection of leukemia using DL and ML techniques is a challenging task. Das et al. (45) present a systematic review of recent advancements in DL- and ML-based ALL detection. The review categorizes various AI-based ALL detection approaches into signal and image processing-based techniques, conventional ML-based techniques, and DL-based techniques, including supervised and unsupervised ML and CNN, recurrent neural network (RNN), and autoencoder-based classification methods. Furthermore, the review categorizes CNN-based classification schemes into conventional CNN, transfer learning, and other advancements. The article provides a critical analysis of recent research, discussing the merits and demerits of the different approaches and highlighting the challenges and future research directions in this field. Overall, this systematic review provides a comprehensive understanding of DL- and ML-based ALL detection, which may assist researchers in formulating new research problems in this domain.

Das et al. (46) propose an efficient deep convolutional neural network (DCNN) framework for accurate diagnosis of ALL, a challenging task. The framework features depthwise separable convolutions, linear bottleneck architecture, inverted residual, and skip connections. It uses a probability-based weight factor to efficiently hybridize MobilenetV2 and ResNet18, preserving their benefits. The approach achieves the best ACC in ALLIDB1 and ALLIDB2 datasets, with superior performance compared to transfer learning-based techniques.

In the field of biomedical image processing, DCNNs have received a lot of attention for various detection and classification tasks. The outcomes of many of them are comparable to or even superior to those of radiologists and neurologists. However, the need for a large dataset makes using such DCNNs difficult to achieve decent results. Paul et al. (47) present a novel single model-based strategy for classifying brain tumors on a short dataset. To avoid overfitting, a modified DCNN known as the RegNetY-3.2G is coupled with regularization DropOut and DropBlock. Additionally, to mitigate the issue of tiny datasets, the RandAugment is an improved augmentation technique. Last but not least, the MWNL (Multi-Weighted New Loss).

Many studies have been introduced using DL techniques for the classification of leukocytes because of the outstanding performance of DL methods for the classification of medical images. The grid search (GS) and random search (RS) hyperparameter optimization methods were used by Hosseini et al. (20) to categorize images of four different categories of leukocytes. ACC of 99% on the training set and of 97% on the validation set was effectively obtained by the given hybrid technique. Through this study (21), the authors highlight the potential of DL, specifically CNNs, in automating the classification of different types of WBCs based on microscopic images. The use of CNNs allows for the detection of significant features that help distinguish different classes of leukocytes, which can assist hematologists in diagnosing diseases such as AIDS and leukemia. The study applied genetic algorithms to optimize the CNN’s hyperparameters and trained the model on a dataset containing 9,957 images and tested it on another dataset of 2,478 images. The optimized CNN achieved high classification ACC, sensitivity, and specificity, indicating its potential as a substitute for manual WBC counting by pathologists. Overall, this study demonstrates the potential of DL techniques in the field of hematology and medical diagnosis. By automating the classification of WBCs, it could lead to more efficient and accurate diagnoses, ultimately improving patient outcomes. CNNs have been presented by Liang et al. (22). This approach can help to strengthen the explanation of input images and discover the structured features of images, and it can also begin end-to-end training of leukocyte images. In particular, they implemented the transfer learning method in order to transfer the feature weights to the CNN segment. Additionally, they implemented a configurable loss function in order to enable the network to train and converge at a faster rate and with more precise parameterization. The findings of their experiments demonstrated that their proposed model for the network has achieved an ACC of 90.8%. The optimized CNN achieved a classification ACC of 99% on the training set, which was 91% for the validation set. In (23), Bairaboina et al. present a DL model developed to classify mature and immature WBCs from peripheral blood smear images. Traditional methods of manual classification by hematologists can be laborious, expensive, and time-consuming. The proposed model uses a combination of W-Net, GhostNet, ResNeXt, and DCGAN-based data augmentation techniques to achieve high ACC levels of 99.16%, 99.24%, and 98.61% for three datasets. The model has potential for clinical application in blood cell microscopic analysis. Another a hybrid approach of recurrent neural networks (RNNs). Leukocyte segmentation was implemented using a network based on W-Net, a CNN-based technique for WBC classification implemented by Rao and Rao (24). Afterward, a DL system based on GhostNet was used to retrieve important feature maps. Then, a ResNeXt approach was used to classify them. The proposed method has attained an ACC of 99.24% on the Blood Cell Count and Detection (BCCD). Rao and Rao (24) presented another DL-based framework for the classification of leukocytes based on the MobilenetV3-ShufflenetV2 DL paradigm. At first, an effective Pyramid Scene Parsing Network (PSPNet) is used to segment the images. When the images have been segmented, the global and local features are extracted and selected using MobilenetV3 and an Artificial Gravitational Cuckoo Search (AGCS)-based technique. Images are then classified into five groups using a ShufflenetV2 model. The proposed method achieves 99.19% and 99% ACC when tested on the BCCD and Raabin-Wbc datasets. Baydilli and Atila (25) have presented a capsule deep neural network (DNN)-based DL system for classifying leukocytes. They have attained an ACC of 96.9% on the benchmarking dataset, LISC. Ghosh and Bhattacharya (26) came up with two distinct models of CNNs that improve and categorize input images of blood cells. On the BCCD benchmarking dataset, they have achieved an ACC of 98.4%. Wang et al. (27) have applied two unique object detection strategies to the problem of leukocyte recognition. These strategies are known as Single Shot Multibox Detector and You Only Look Once (YOLO). In order to enhance the performance of recognition, several essential elements affecting these object detection strategies have been investigated, and detection models have been constructed utilizing a private dataset. The level of ACC that was achieved was 90.09%. Ma et al. (28) have come up with a new framework for the classification of blood cell images. This framework is built on a deep convolutional generative adversarial network (DC-GAN) as well as a residual neural network (ResNet). They have accomplished a level of precision on the BCCD dataset that is 91.7% accurate. By bringing together the ideas of merging the features of the first and last convolutional layers and propagating the input image to the convolutional layer, Banik et al. (29) created a novel CNN model. They additionally employed a dropout layer to mitigate the model’s overfitting issue. On the BCCD test database, they have obtained an average ACC of 96%. Sahlol et al. (30) have used VGGNet, a robust CNN architecture, already trained on ImageNet, to extract features from images of leukocytes. The statistically improved Salp Swarm Algorithm was then used to filter the extracted features. This optimization method takes biological principles as its inspiration, picking the most important features while discarding those that are excessively linked or noisy. ACC of 83.2% was attained when the proposed method was used on the C-NMC public Leukemia reference dataset.

## 3 Materials and methods

### 3.1 Dataset

The BCCD public dataset (25) contains 12,453 augmented images of leukocytes in JPEG format and cell type labels in CSV format. There are 3,120, 3,103, 3,107, and 3,123 augmented images for each class of the four cell types of eosinophil, lymphocyte, monocyte, and neutrophil, respectively, as compared with the 88, 33, 21, and 207 original images (Mooney, 2018). The basophil images are removed from the dataset as that type typically makes up less than 1% of the leukocytes. A drop of blood is placed on a glass slide and smeared with a spreader slide. The blood is stained with a Romanowsky stain such as May-Gr ¨u nwald Giemsa, Wright, or Wright–Giemsa. Image quality, illumination, and different staining techniques affect the outcome. The taken picture of cells is magnified 100× and converted to standard RGB channels. The dataset has been preprocessed, as each image was augmented and repositioned before it is made available to the public for the input of the CNN to avoid overfitting. The BCCD database is split into two sets: approximately 80% of the data (9,966 images) for the training set and 20% (2,487 images) for the validation set. The training set is composed of 2,497, 2,483, 2,487, and 2,499 images of eosinophil, lymphocyte, monocyte, and neutrophil, while the validation set contains 623, 620, 620, and 624 images of eosinophil, lymphocyte, monocyte, and neutrophil.

Neutrophils are the most numerous types of leukocytes constituting 50%–70% of the circulating leukocytes (44). The nucleus is relatively small and often multilobed. The stained nucleus is dark blue, and its CNR is 2:1. They are capable of phagocytizing viruses, toxins, fungi, and bacteria. They are the first line of defense once microbial infection strikes.

Eosinophils compose 1%–5% of the leukocytes; however, their counts fluctuate under different conditions (44). The cytoplasm is pink-stained while the nucleus is purple-stained and frequently is bilobed connected by a band of nuclear material. They protect against parasitic infections and cancer cells. They produce histamine as an inflammatory response to allergy-inducing agents, damaged tissue, or pathogen invasion.

Lymphocytes constitute 20%–45% of leukocytes and are much more common in the lymphatic system than in blood (22). They are agranular cells with a large dark purple-stained nucleus and a relatively small pale-colored amount of cytoplasm (38). They create antibodies to regulate immune system responses against bacteria, viruses, and other potentially harmful agents. The main types of lymphocytes are T cells, B cells, and natural killer cells.

Monocytes make up approximately 2%–10% of leukocytes and are the biggest leukocyte (22). Monocytes are granular and have a kidney-shaped nucleus with plenty of light blue cytoplasm. They share the phagocytic ability of neutrophils, break down bacteria, and remove waste from the blood. They have a longer life span compared with other leukocytes (20).

The BCCD database is augmented before becoming publicly available on the Kaggle website because, practically, the amount of training data is usually limited or not sufficient. Augmentation expands the training set with artificial data so it can be used by researchers. For the classification tasks, that means receiving a high-dimensional input such as images and producing a related output. A good classifier is immune to a wide-ranging variation. CNN as a framework well-established for image data can discriminate relevant minor features in the image while it is invariant to unrelated large variations in the image (26). For image datasets, augmentation can be done by modifying the images a few pixels to improve the generalization ability and avoid overfitting. Among available transformations are flipping, scaling, zooming, and rotating the image in several directions. Augmentation helps increase the correct classification rate regardless of size, position, or degree of distortion of an image. Using random transformation exposes the network to more features in the data so it can generalize better. One thing to consider when using an augmentation approach is that one should take care of not altering the correct class by using the wrong transformations (42, 44).

### 3.2 Convolutional neural network and transfer learning

The CNN model is made up of multiple layers, including an input layer, convolutional layers, batch normalization layers, pooling layers, ReLU layers, Softmax layers, and one output layer. The dimensions a, b, and c of the input image make up what is known as the input layer. The total number of channels is specified by c. The main and first convolutional layer of the network takes in data via three separate inputs labeled a, b, and c. The convolutional layer is the one that is responsible for mapping out the features. The activation layer makes use of these features, which are also put to use for visualization purposes. Transfer learning makes use of an already trained and reused model as the foundation for a new task and model. The model used for one task can be repurposed for other tasks as an optimization to improve performance. By applying transfer learning, the model can be trained with a small volume of data. It is helpful to save time and achieve good results. In the transfer learning approach, we transfer knowledge from the source mammogram input images to the target domain mammogram mass images IT. The target classifier Tc (Mt) is to be trained from the input mammogram image Is to the target image IT to get the classifier prediction about BMNTi, which stands for benign, malignant, and normal. To extract the features, a transfer layer is used. The top layer from the classifier retrained the new target classes, while the other layers were kept frozen as defined in Equation 1.


(1) $$BMN_{Ti}=T_{c}\left(M_{t}\right)$$

#### 3.2.1 DenseNet

**Figure 3:**
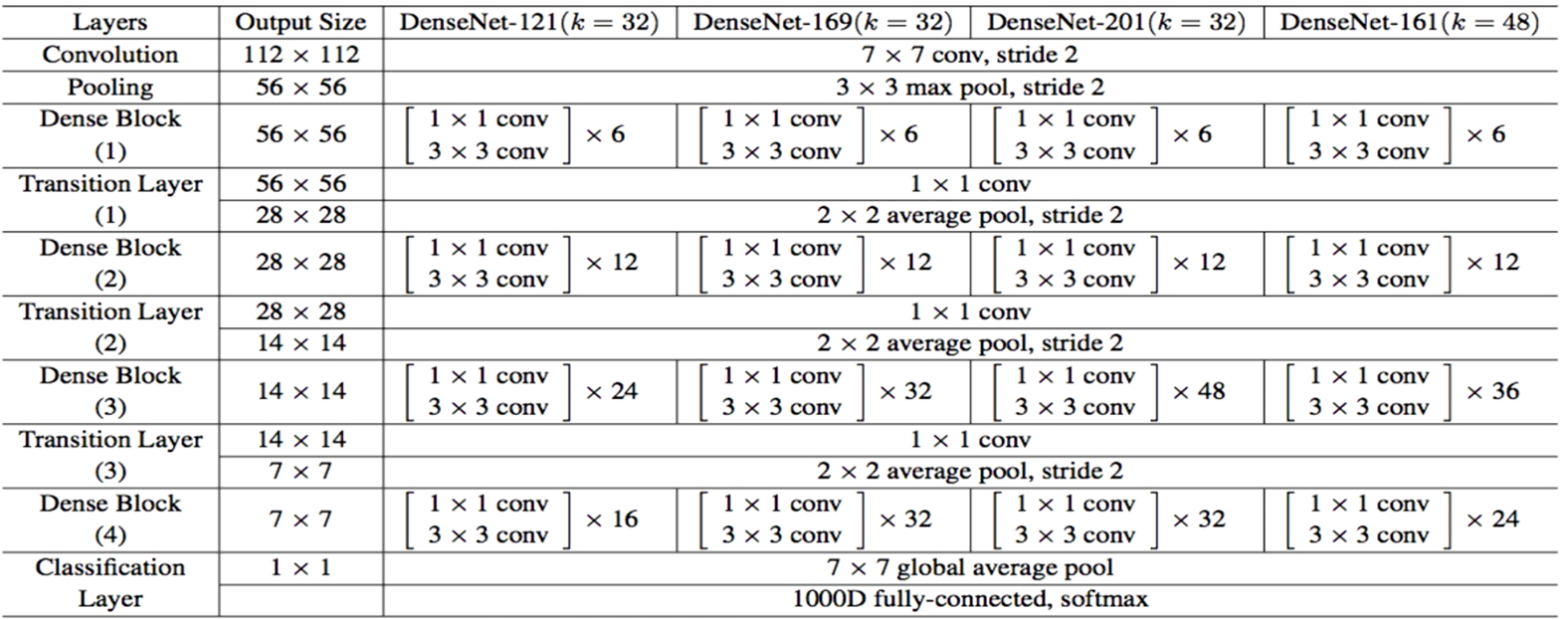
DenseNet architectures for ImageNet. DenseNets are broken up into DenseBlocks, and while the dimensions of the feature maps stay the same inside each block, the number of filters that are used varies from one block to the next. These layers in between them are referred to as Transition Layers.

DenseNets are the subsequent stage to increase the depth of deep convolutional networks. When CNNs go deeper, the problems arise. This happens because of the big path for information from input to output layer. DenseNet-161 is a simple connectivity pattern because it connects all layers directly with each other to be sure that information flow is maximum between layers in the network. Feed forward nature is maintained by obtaining each layer additional inputs from the preceding layers. **Figure 3** presents the architectures of DenseNet for ImageNet. Features are combined by concatenation. DenseNet is not as the same as traditional architecture because it introduces $\frac{L\left(L+1\right)}{2}$ connections in an L-layer network in lieu of L. Handling problems of vanish gradient, reusing feature, lacking parameter’s number, and propagating features is the most important feature of DenseNets.

#### 3.2.2 Mathematical model of DenseNet deep networks

DNNs have reached state-of-the-art performance in a variety of computer vision applications. Moreover, the interpretation of DNNs has been examined from the perspective of visualization as well as resilience. The groundbreaking studies that highlight the potential of DNNs include AlexNet and VGGNet. The community’s research focus has changed from feature engineering to network design engineering as a direct result of the success of these key efforts. As a result, various new network architectures have been developed to improve the performance of DNNs. ResNets have achieved state-of-the-art performance on a variety of benchmark datasets, including ImageNet and the COCO detection dataset. This was accomplished by reusing previous features in conjunction with the identification shortcut. One of the factors that contribute to ResNet’s phenomenally high level of popularity is its straightforward design strategy, which includes just one identity shortcut. The shortcomings of the identity shortcut have been investigated in subsequent publications, despite the tremendous success that it has enjoyed. Because the identity shortcut bypasses the residual blocks to maintain characteristics, it is possible that the network’s capacity for representation is diminished as a result.

**Figure 4:**
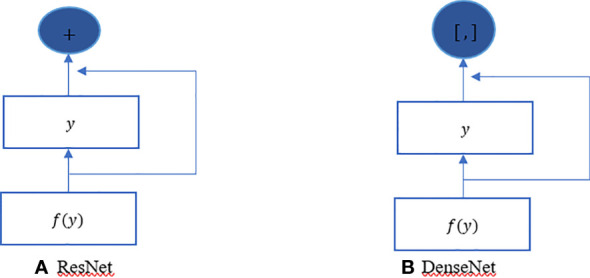
DenseNet vs. ResNet. The primary distinction between **(A)** ResNet and **(B)** DenseNet is that, in the latter case, outputs are concatenated (shown by) instead of added.

**Figure 5:**
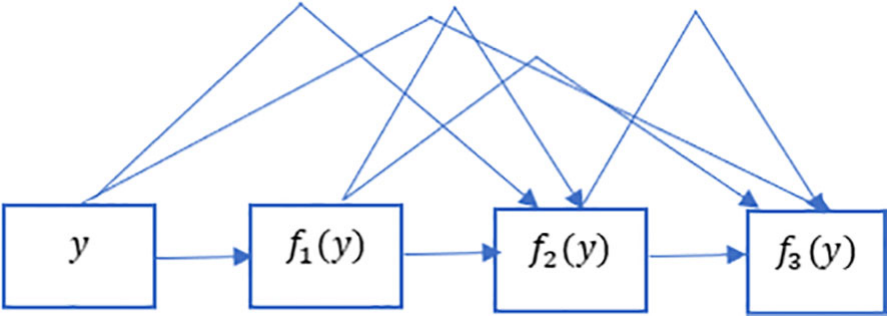
Dense links in DenseNet. DenseNet gets its name from how packed the dependency tree between the variables gets. The last layer in this structure has numerous connections to its predecessors. This figure depicts these complex interconnections.


The ResNet has brought about a fundamental shift in how it was thought to parametrize the functions of DNNs. The DenseNet can be thought of as a kind of logical extension of this. Both the connection pattern in which each layer connects to all the preceding layers and the concatenation operation (as opposed to the addition operations in ResNet) to retain and reuse features of previous layer are defining characteristics of the DenseNet architecture. Let us make a brief detour into mathematics to comprehend how one might possibly arrive at such a conclusion. Looking back to functions’ Taylor expansion. To clarify, for a point y=0, it might be expressed as shown in Equation 2. One of the most important features of ResNet is that it can break down a function into a series of terms with progressively higher orders. In a manner analogous to this, ResNet disassembles functions, as demonstrated in Equation 3. In other words, the ResNet breaks down a function *f* (y) into a straightforward linear component and a complex nonlinear one. However, if we were to write down more information than just the two components, but not necessarily add anything new. DenseNet is one example of such a solution. **Figure 4** illustrates the primary difference between ResNet (shown on the left) and DenseNet (shown on the right) in terms of cross-layer connections: the utilization of addition versus the utilization of concatenation. As can be seen in **Figure 4**, the primary distinction between ResNet and DenseNet is that, in the latter case, outputs are concatenated (shown by) instead of added. This is denoted by the notation. As a consequence of this, we apply an increasingly complex chain of functions before performing a mapping from the values it contains, as depicted in Equation 4. The number of features is further reduced by combining all of these functions in Multi-layer Perceptron (MLP). The mechanics of this are straightforward; instead of adding, we just string together the terms. DenseNet gets its name from how packed the dependency tree between the variables gets. The last layer in this structure has numerous connections to its predecessors. **Figure 5** depicts these complex interconnections.

(2) $$f\left(y\right)=f\left(0\right)+y\;.\;[\;f^{\prime }\left(0\right)+y.[\;\frac{f^{\prime \prime }\left(0\right)}{2!}+y\;.\left[\;\frac{f^{\prime \prime \prime }\left(0\right)}{3!}+\ldots \right]]\;]$$

(3) f(y)=y+g(y) 

(4) $$y\;\to \left[y,\;f_{1}\left(y\right),\;f_{2}\left(\left[y,\;f_{1}\left(y\right)\right]\right),\;f_{3}\left(\left[y,\;f_{1}\left(y\right),\;f_{2}\left(\left[y,\;f_{1}\left(y\right)\right]\right)\right]\right),\;\ldots \right]$$

### 3.3 The proposed CAD system for leukocyte images

**Figure 6:**
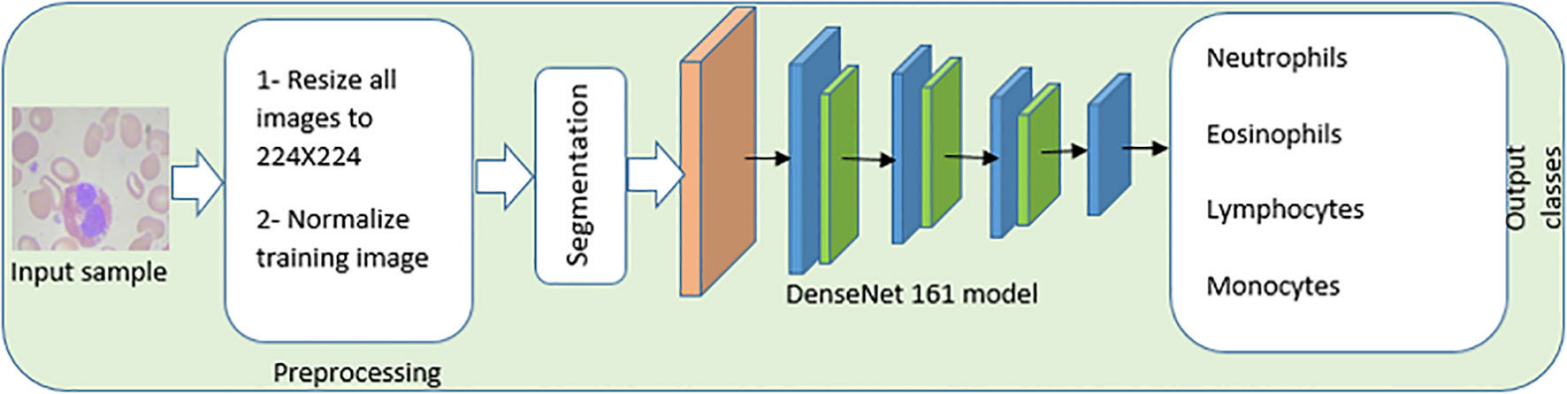
An automated End-to-End CAD, system of white blood cells. The image preprocessing and enhancement stage, the image segmentation stage, the feature extraction and selection stage, and the white blood cell classification stage are the four primary stages that are included in the proposed CAD system.

The image preprocessing and enhancement stage, the image segmentation stage, the feature extraction and selection stage, and the WBC classification stage are the four primary stages that are included in the proposed CAD system. These stages are illustrated in **Figure 6**, which also contains the information that is mentioned in the *Introduction* section. In addition, the findings of this research contribute to existing state-of-the-art models by suggesting the implementation of a one fit cycle strategy, which makes the process of training simpler. As a result, there is no requirement to adjust any of the hyperparameters of the network that is being used.

#### 3.3.1 Image preprocessing phase

In order to process the input histopathological image sample, images are resized to 244 × 244, and training images are the only ones that are normalized. Changing the range of intensity values for individual pixels is the core idea behind image normalization. The purpose of image normalization is to transform the pixel range values into ranges that are more intuitive to the senses.

#### 3.3.2 Image segmentation phase

**Figure 7:**
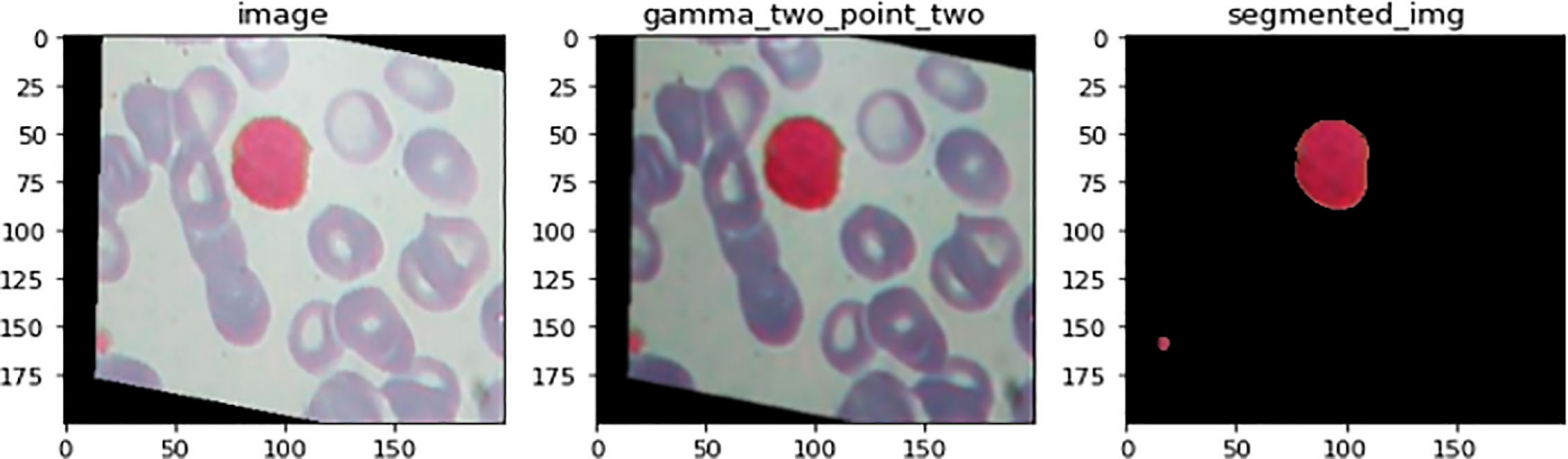
An illustration of image before and after the segmenation phase. This is an example for an input image with its corresponding output image, segmented one.

**Figure 8:**
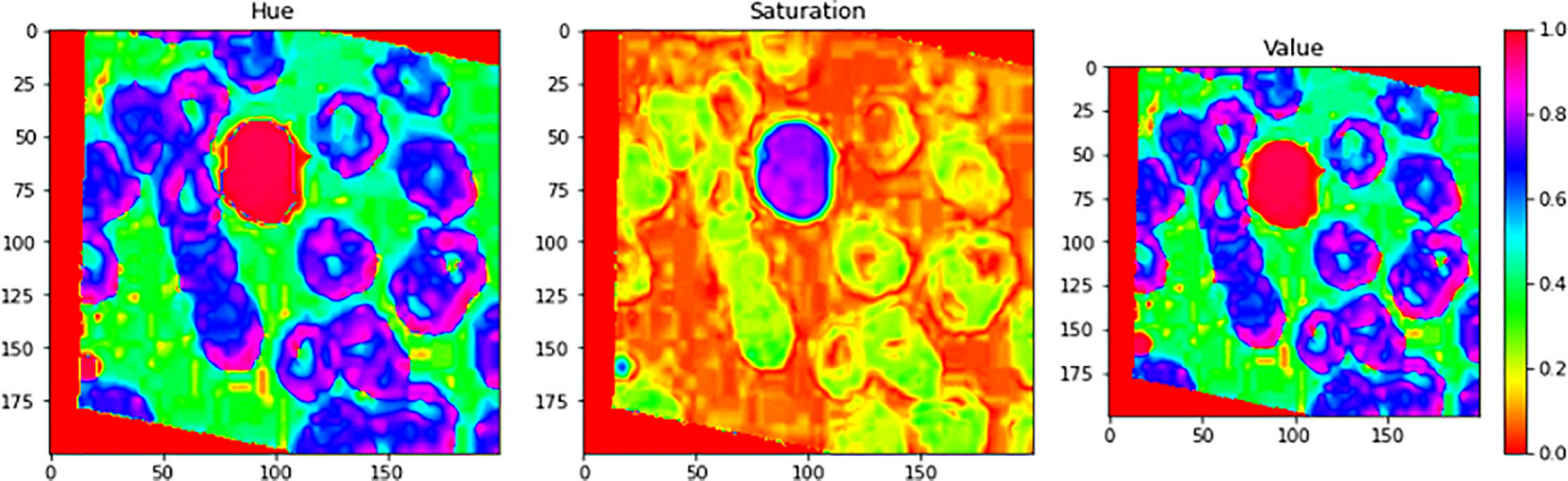
Color Image Segmentation was used on the images to separate each individual pixel using the HSV color space. The images will be segmented using information derived from the HSV color space. HSV is an abbreviation that stands for Hue, Saturation, and Value.

**Figure 7** illustrates an example for an input image with its corresponding output image, segmented one. Color Image Segmentation was used on the images to separate each individual pixel using the HSV color space. The images will be segmented using information derived from the HSV color space. HSV is an abbreviation that stands for hue, saturation, and value as illustrated in **Figure 8**.

The following **Algorithm 1** is an outline of the primary steps that are involved in the image segmentation phase:

Algorithm 1 **Image segmentation phases.**


i) First, convert the RGB image in HSV form as depicted in 
**Figure 6**.
ii) using the color bar at the right to choose the
thresholds.
iii) set up the thresholds for the masks. 
 • Lower Mask (refer to the hue channel)
 • Upper Mask (refer to the hue channel)
 • Saturation Mask (refer to the transparency
   channel)
 Ex: to segment the NEUTROPHIL cell, the lower and upper 
 mask values that are appropriate would be 0.0 and 1.0
 After that, the saturation threshold is decided. This
 is a bit tricky because you need to consider the colors
 that are seen in the object. In this case, the values 
 are 0.45
iv) Create the mask by multiplying all masks of the 
 thresholds.
 mask = upper_mask*lower_mask*saturation_mask
v)Then, multiply this mask by each value in the rgb
 image.
 red = img [ : , : , 0 ]*mask
 green = img [ : , : , 0 ]*mask
 blue = imh [ : , : , 0 ]*mask
i) Lastly, apply the morphology operation to remove the 
 noise or halls.

#### 3.3.3 Feature selection and classification using the DenseNet model

The DenseNet-161 DL model is used in the implementation of both the feature extraction and classification stages. Adjouadi et al. (34) developed DenseNet that had the best classification results on the available datasets such as ImageNet. DenseNet does not use direct connections among hidden layers, but it uses dense connection to build a model. Its construction was based on linking each to a subsequent layer. In any layer, any important features learned are involved within the network. Due to the extracted features, deep network training became more efficient and the performance of the model increased. The number of parameters has become less than CNN because feature maps are sent directly to all subsequent layers. The DenseNet has a very important feature, which is that it reduces overfitting in the model because of the use of dense connections. Training DL models with very large numbers of parameters takes much time. More and more data and powerful GPU are required to train these models from scratch. Transfer learning is used to overcome the pervious problem. By using transfer learning, you are saving time. Transfer learning is a method of machine learning in which a model that was developed for one task can be utilized as a foundation for a model that will be used on a different task. Learned features are often transferable to different data. For example, a model trained in Dataset for animal images that includes learned features such as edges and lines can be used on other dataset using transfer learning technique. In transfer learning, feature extractor is done by fully connected layer after removing it from the model used.

In this study, DenseNet-161 with ImageNet is utilized; however, the final layer, which is designated as the “completely connected” layer, has had its number of classes reduced from 1,000 to 4. The strategy known as one fit cycle policy is utilized to implement DenseNet-161.

#### 3.3.4 One fit cycle policy

**Figure 9:**
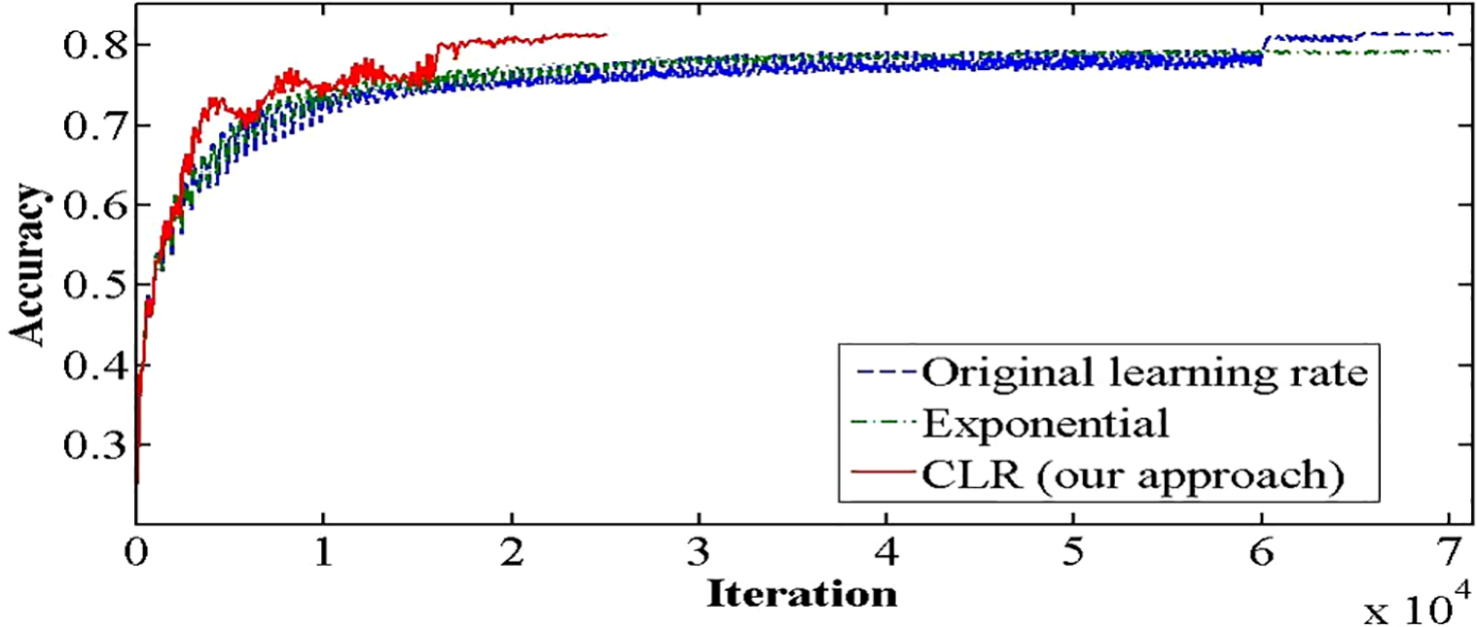
CLR method and original learning rate.

It is known that training of DNN is a difficult optimization problem. Tuning of hyperparameters such as LR is very important. The performance of the network will be enhanced by carefully selecting the hyperparameters for LR, momentum, and weight decay. The traditional approach involves running a grid or random search, which can be time-consuming and computationally intensive. The impacts of these hyperparameters are also closely related to the architecture, the data, and each other. This section provides more effective guidelines for selecting certain hyperparameters (27). A small LR leads to very slow training, while a large LR hinders the convergence. A low LR is good, but it takes a long time to train perfectly. When training speed is increased, LR is increased until LR gets too large and diverge. To obtain the exact LR, you need to do many experiments and be patient. A new method was discovered by Leslie N. Smith for setting up LR named CLRs. CLR made LR values between minimum and maximum range instead of having fixed values during the training. CLR cycle has two steps, one of them being an increase in LR and the other one being a decrease in LR. CLR eliminates the need to find the optimal LR but the optimal rate between minimum and maximum range. **Figure 9** shows classification ACC while training CIFAR-10. The red curve is CLR. As depicted in **Figure 9**, the CLR achieves the same ACC as the original LR but in iteration less than the original LR method (15). In Leslie N. Smith’s research (48), super-convergence is the method that uses CLR, but with one cycle that contains two LR steps. The total number of iterations must be larger than the size of the cycle. After completing the cycle, LR is decreased much further for the remaining iterations. Leslie N. Smith named this method one fit cycle policy. In super-convergence, LR starts from a small value and is increased to a very large value then returns to a value lower than its initial one. The impact of LR many values is a training ACC curve. In super-convergence, training ACC is moved fast as LR is increased (15, 44), becomes oscillated as LR is very large, and then jumps again to an extreme point of ACC.

To utilize CLR, one must provide a step size and minimum and maximum LR bounds. A cycle consists of two such steps, one in which the LR linearly grows from the lowest to the maximum and the other in which it progressively falls. The step size is the number of iterations (or epochs) utilized for each step. Smith (2015) explored a variety of methods for varying the LR between the two boundary values, discovered that they were all equivalent, and thus advised the most straightforward method—letting the LR change linearly—even though suggested discrete jumps and found similar outcomes (28).

Training for the LR range test begins with a modest LR and gradually rises linearly over the course of a pretraining run. This single run offers useful insight into the maximal LR and how well the network can be taught over a variety of LRs. The network starts to converge at a low LR, and as the LR rises, it finally reaches an unmanageable size, which lowers ACC and increases test/validation loss. By using a constant LR, a smaller number is required since otherwise the network will not start to converge. The LR at these extrema is the highest value that can be utilized as the LR for the maximum bound with CLRs. The minimal LR constraint can be chosen in a variety of ways: 1) by a factor of 3 or 4 less than the maximum bound, 2) by a factor of 10 or 20 less than the maximum bound if only one cycle is used, or 3) by a quick test of hundreds of iterations with a few initial LRs and choosing the largest one that permits convergence to start without overfitting. If the initial LR is too large, the training will not start to converge. Be aware that the LR can only rise to a certain point before the training becomes unstable. This affects your decision about the lowest and maximum LRs (i.e., raise the step size to widen the gap between the minimum and maximum).

## 4 Results and discussion

The experiments are applied on a BCCD public dataset. Our studies were carried out on it with the help of Google Colab. The evaluation criteria are used to evaluate the performance of classification model, including image test ACC, Macro-F1, Micro-F1, and Kappa criteria, and average time. Macro-F1 takes the average of the precision and recall of each class. ACC is defined by the ratio of Ncor (the number of correctly classified images in testing set) to Nall (Total number of images in testing set). Equation 5 defines the image test ACC. Precision is calculated as the sum of true positives across all classes divided by the sum of true positives and false positives across all classes. Recall is calculated as the sum of true positives across all classes divided by the sum of true positives and false negatives across all classes. Equation 6 defines Micro-F1. Kappa measure, based on confusion matrix calculation, can handle problems such as imbalanced datasets and multiclass problems. Precision is defined by Equation 7, and it means the percentage of your results that are relevant. On the other hand, recall as described by Equation 8 refers to the percentage of total relevant results correctly classified by your algorithm. Equation 9 defines Kappa coefficient, where $p_{0}$ is the image test ACC as defined in Equation 5, and $p_{e}$ is the summation of the product of the number of images in each type of cancer and the predicted number of images in each type of cancer to the square of the total number of images in the testing set.


(5) $$Accuracy\left(ACC\right)=\frac{\text{N}cor}{N_{all}}$$

(6) $$F1-score=\frac{2X\left(precision\;\times \;recall\right)}{\left(precision+\;recall\right)}$$

(7) $$Precision=\frac{\text{Sum c in C TruePositives}\_\text{c}}{\text{Sum c in C }\left(\text{TruePositives}\_\text{c }+\text{ FalsePositives}\_\text{c}\right)}$$

(8) $$Recall=\frac{\text{Sum c in C TruePositives}\_\text{c}}{\text{Sum c in C }\left(\text{TruePositives}\_\text{c }+\text{ FalseNegatives}\_\text{c}\right)}$$

(9) $$Kappa=\begin{array}{ccc} \frac{p_{0}-p_{e}}{1-p_{e}}, & p_{0}=\frac{\text{N}cor}{N_{all}}, & p_{e} \end{array}=\frac{\sum^{}\text{N}trueX\text{N}pre}{N_{all}XN_{all}}$$

### 4.1 Classification results

**Figure 10:**
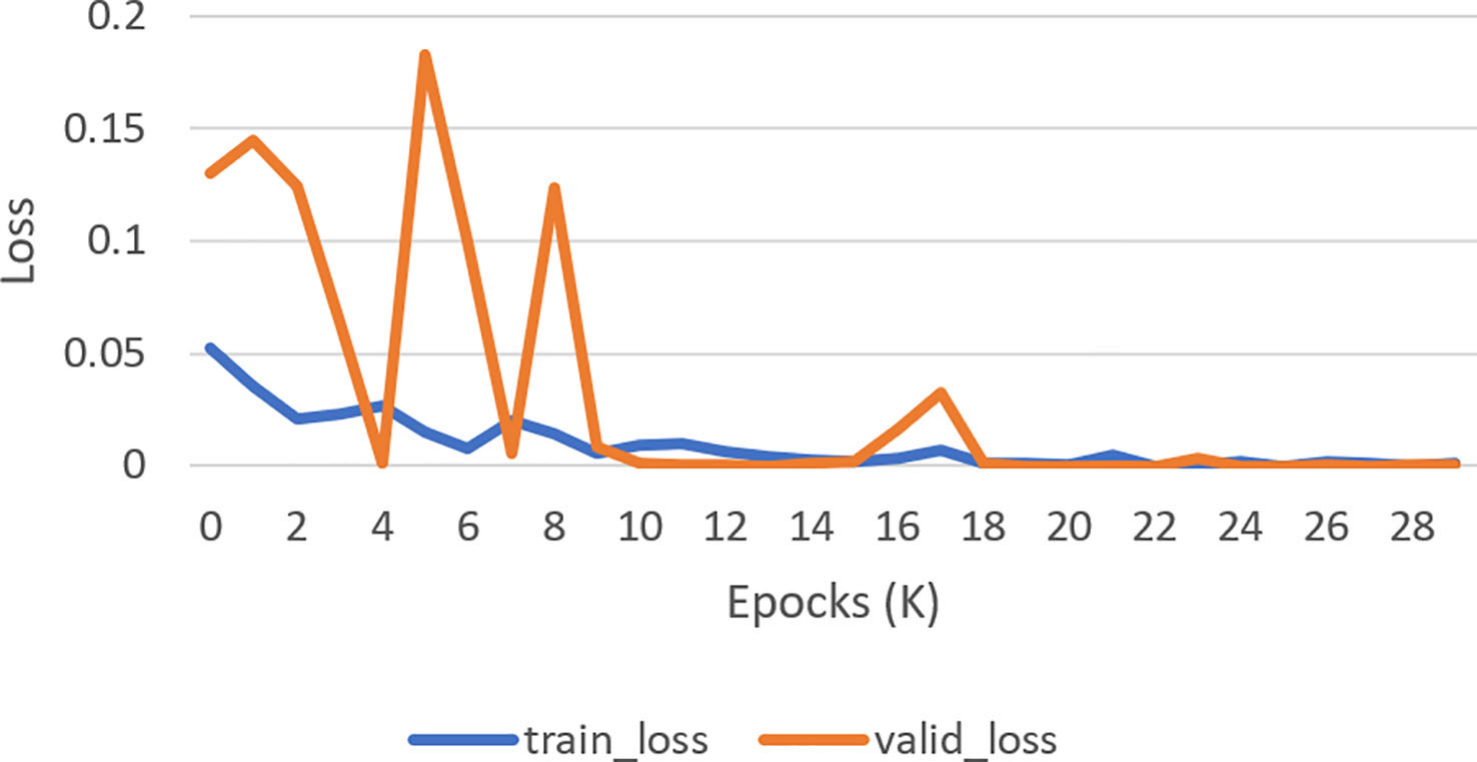
Loss curve. How well a model matches its training data is measured by the validation loss (Orange curve), whereas how well it performs on novel data is measured by the training loss (Blue curve).

**Figure 11:**
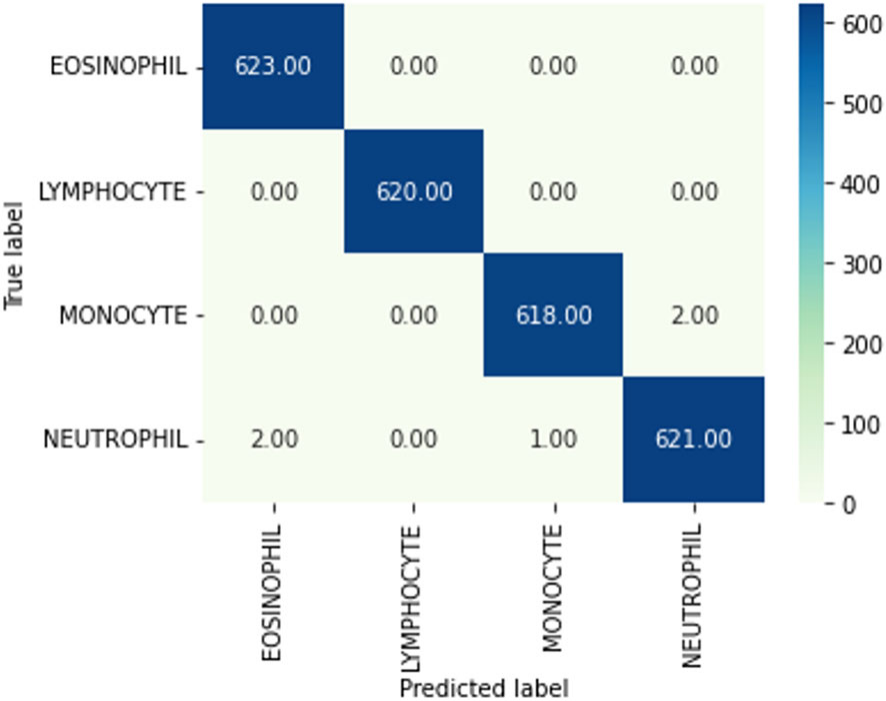
Confusion matrix. A confusion matrix is a graphical representation and summary of a classification algorithm's results. There is a one hundred percent rate of accuracy for classifying Lymphocyte and Eosinophil samples. There are two Monocyte samples that have been mislabeled as Neutrophils.

The next subsection discusses the classification result on the BCCD dataset based on the default one fit cycle policy approach. The experimental result is applied on a raw dataset. Moreover, the results of our research experiments are compared with the results of other researchers. The experiments are performed over a desktop computer system having an Intel Core i7-7700 CPU, 16 GB RAM, and one 8-GB GPU. This research used DenseNet-161 to perform the classification of microscopic images into neutrophils, eosinophils, lymphocytes, and monocytes by using a pretrained model in terms of ACC, F1, AUC, and Kappa. Our experimental result of multiclassification problem on raw data is shown in **Table 1** according to ACC, Macro-F1, Micro-F1 and Kappa. We ran the raw data on 30 epochs. All classification results are given in **Tables 2**, **3**. The loss curves are shown in **Figure 10**, and the confusion matrices are shown in **Figure 11**.

**Table 2:** The result of each evaluation is on raw data.

Network	Criteria	Result	Average Time
DenseNet-161	Accuracy (ACC)	0.985	4.30 h

**Table 3:** Precision, Recall, and F1-score for raw data.

Criteria	Types	precision	recall	f1-score
	Eosinophil	1.00	1.00	1.00
	Lymphocyte	1.00	1.00	1.00
	Monocyte	0.80	1.00	0.89
	Neutrophil	1.00	0.98	0.99
accuracy	–	–	–	0.99
macro avg	–	0.95	0.99	0.99
weighted avg	–	0.99	0.99	0.99

The experimental results in **Table 3** show that all evaluation metrics on 40× magnification factor (which is indicated by the black underline) are better than the other magnification factors. The reason for 40× achieving the best ACC is because it contains more significant features of breast cancer. From **Table 3**, precision, recall, and F1-score values show that our model classification result is perfect.

**Figure 12:**
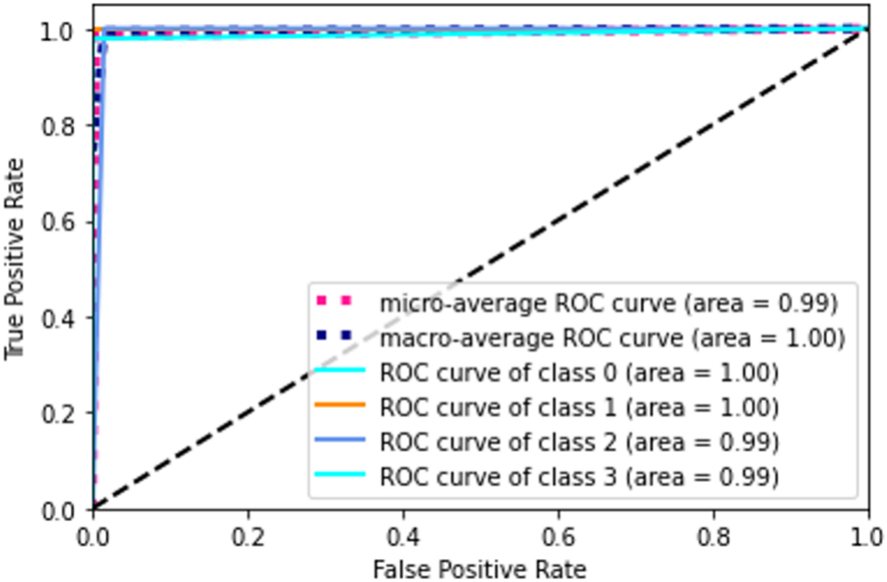
Confusion matrix. A confusion matrix is a graphical representation and summary of a classification algorithm's results. There is a one hundred percent rate of accuracy for classifying Lymphocyte and Eosinophil samples. There are two Monocyte samples that have been mislabeled as Neutrophils.

The receiver operating characteristic (ROC) metric is used to evaluate the output quality. ROC is a probability curve, while AUC, area under the curve, is a metric for assessing how well two groups may be distinguished. It reveals the extent to which the model can differentiate between categories. If the AUC is high, then the model is very good at predicting the correct classes. The AUC value (see in **Figure 12**) for class 0 and class 1 is 1.00 and for class 2 and class 3 is 0.99. Ideally, the ROC for the false positive rate should be zero and one for the true positive rate.

### 4.2 Comparisons with other models

This section compares our experimental results with the other experiments carried out by other research papers on raw data and augmented data. We evaluated the ACC of the newly introduced method to that of the most recent and cutting-edge classification frameworks for leukocyte histology by using the BCCD database. As a consequence of this, we were in a position to evaluate the significance of the hybrid DenseNet and CLR approach. The differences between the suggested method and the state-of-the-art methods that are currently in use are outlined in **Table 4**. To facilitate this comparison, ACC was utilized as a performance metric.

**Table 4:** A comparison between research results and the state of the art.

Criteria	Author	Methods	Result
Accuracy (ACC)	Maryam et al. (20)	Optimized CNN	99%
	Bani-Hani et al. (21)	GA-optimized CNN	91%
	Liang et al. (22)	Hybrid CNN-RNN	90.8%
	Rao (23)	CNN and ResNeXt	99.24%
	Rao (24)	ANN and CNN	97.7%
	Ghosh and Bhattacharya (25)	CNN and FCN on noise-free cell images	98.4%
	Ma et al. (26)	DC-GAN, and ResNet	91.7%
	Banik et al. (27)	CNN	96%
	**Proposed model**	**DenseNet-161 with CLR Approach**	**99.8%**

DenseNet with the CLR approach, the suggested classification framework, outperforms the DL systems established by Bani-Hani et al. (40), Liang et al. (22), Paul et al. (47), Bairaboina and Battula (23), Rao and Rao (24), and Banik et al. (29) when applied to the BCCD dataset. In addition to this, it has accomplished a level of ACC that is on par with that which Habibzadeh et al. (39), Rao and Rao (24), and Ghosh and Bhattacharya (26) have accomplished.

In general, it can be deduced from the comparison in **Table 4** that the suggested system is capable of recording a performance that is better than that of all other systems.

From the experimental results applied on raw and augmented data, **Tables 2**–**4** show that the evaluation criteria-specified ACC achieved the best results by applying a new method in training called one fit cycle policy and with small number of batches and the fewest number of epochs. When we have trained the CNN using 32 batch size and 60 epoch, we did not attain high performance. On the contrary, we use 32 batch sizes and 30 epochs on raw data, and this helped us to reduce the time of training and achieve better ACC than the other research.

## 5 Conclusions

Using a combination of the recently developed pretrained CNN, DenseNet, and the one fit cycle policy, this study describes a technique of training for the classification of WBCs. The proposed method is more accurate and requires less cycles to train CNN—thanks to the one fit cycle policy. It fixes how difficult it is to adjust the DL model’s hyperparameters. DenseNet-161 was used in the experiment, and the results are analyzed in terms of various performance indicators. ACC, precision, and recall are presented as indicators of the suggested model’s efficacy. We solved the multiclass classification problem with a raw data ACC of 99.8%. As a result, the outcomes of our experiments are more reliable than those obtained in the existing state of the art for the classification of WBCs. In the future work, the proposed model can be applied to diagnosis-specific diseases such as cancer and liver disease.

## Data availability statement

The original contributions presented in the study are included in the article/supplementary material. Further inquiries can be directed to the corresponding author.

## Author contributions

Supervision and methodology, EH; software, EH and OM; conceptualization, EH, NS, RT, AA-H, RA-T, and NM; validation, EH, NS, RT, AA-H, RA-T, and NM; formal analysis, EH and OM; investigation, EH and OM; resources, NS and NM; data curation, NS and NM; visualization, EH, OM, NS, RT, AA-H, RA-T, and NM; writing—original draft preparation, OM; writing—review and editing, EH, OM, NS, RT, AA-H, RA-T, and NM; funding acquisition, NS and NM. All authors discussed the results and approved the final paper. All authors have read and agreed to the published version of the article.
